# Development of an Innovative Non-Destructive and Field-Oriented Method to Quantify the Bond Quality of Composite Strengthening Systems on Concrete Structures

**DOI:** 10.3390/ma13235421

**Published:** 2020-11-28

**Authors:** Astrid Billon-Filiot, Frédéric Taillade, Marc Quiertant, Jean-Marie Hénault, Jean-Claude Renaud, Romain Maurin, Karim Benzarti

**Affiliations:** 1 Electrotechnique et Mécanique des Structures (ERMES) Department, R&D Division, Electricité de France (EDF), 91120 Palaiseau, France; 2 Performance, Risque Industriel et Surveillance pour la Maintenance et l’Exploitation (PRISME) Department, R&D Division, EDF, 78400 Chatou, France; frederic.taillade@edf.fr (F.T.); jean-marie.henault@edf.fr (J.-M.H.); 3 Matériaux et Structures (MAST) Department, Université Gustave Eiffel—Institut Français des Sciences et Technologies des Transports, de l’Aménagement et des Réseaux (IFSTTAR), 77454 Marne-la-Vallée, France; marc.quiertant@ifsttar.fr (M.Q.); jean-claude.renaud@ifsttar.fr (J.-C.R.); 4 Matériaux et Mécanique des Composants (MMC) Department, R&D Division, EDF, 77250 Ecuelles, France; romain.maurin@edf.fr; 5 Laboratoire Navier, Université Gustave Eiffel—Ecole Nationale des Ponts et Chaussées (ENPC), Centre National de la Recherche Scientifique (CNRS), 77447 Marne-la-Vallée, France; karim.benzarti@univ-eiffel.fr

**Keywords:** FRP composite strengthening systems, concrete structures, bond quality, nondestructive evaluation, pull-off test, distributed optical fiber sensing

## Abstract

Over the last 30 years, structural reinforcement and retrofitting with externally bonded composite materials have proven to be efficient and cost-effective solutions to increase both the safety and the lifespan of civil engineering structures, including nuclear power plants. The effectiveness of the strengthening system highly depends on the level of adhesion between the fiber-reinforced polymer (FRP) composite material and the concrete surface. Therefore, on-site evaluation of the bond quality is critical to assess the performance and predict the durability of the system in place. The direct tension pull-off test is most commonly used to quantify the adhesion level, but this standardized method has many drawbacks. In the present study, it is proposed to evaluate the bond properties by using a nondestructive test (NDT) derived from the standard pull-off test. This innovative test enables the measurement of an interfacial “stiffness” which may be used as a bond quality criterion. This paper gives an insight into the performance of the proposed NDT method, when applied in laboratory conditions to concrete slabs reinforced with bonded pultruded carbon FRP plates (CFRP). Three different epoxy adhesive systems with a broad range of Young’s moduli were used for the specimens’ preparation, in order to vary the stiffness of the concrete/CFRP interface. The purpose was to simulate different levels of interfacial adhesion that could be observed for a single adhesive system. It was shown that the test method was able to detect differences in the interface stiffness beyond experimental uncertainties, and it should therefore enable the detection of differences in the bond quality for a given adhesive system as well. The sensitivity of the NDT was then discussed, and its detection capabilities were predicted for standard field conditions. In the last part, strain measurements were collected during the NDT, thanks to distributed optical fiber sensors (DOFS) embedded in the adhesive joints of the strengthened specimens. An analysis of the strain profiles was found to provide complementary information on the quality of the adhesive bond.

## 1. Introduction

For civil engineering applications, reinforcement systems based on externally bonded fiber-reinforced polymer (FRP) materials are now commonly used [1,2,3]. These composite materials are either installed by wet lay-up process or by bonding pultruded plates with epoxy adhesives. In the concrete−adhesive−FRP assembly, stress is transferred from the host concrete structure to the composite material through shear deformation of the adhesive layer. Therefore, both efficiency and durability of the strengthening system depends on the quality/integrity of the FRP-to-concrete adhesive joint.

A high quality of adhesion implies, obviously, that the bond layer does not present macroscopic defects like voids or cracks, which may initiate critical flaws and lead to the premature failure of the assembly. Such defects can be detected using simple nondestructive test (NDT) methods like hammer inspection, or by more sophisticated methods involving mechanical-wave-based techniques (impact-echo [4], acoustic emission, ultrasonic inspection [5] and impedance measurements [6]) or imaging techniques (X-ray tomography [7], infrared thermography [8], laser reflection [9] and shearography [10]). A classification of NDT methods used to detect, localize and quantify defects in the case of FRP systems bonded to civil engineering structures can be found in [11].

However, a high bond quality not only implies that the FRP-to-substrate interface is free of defects like voids and cracks, but also requires that the material and interfacial properties of the adhesive layer are sound. It is therefore of primary importance to assess that these properties are optimal, or at least at a sufficient level, so that the FRP reinforcement fulfills guideline specifications at the time of the strengthening works, and over its design service life. 

A common approach for assessing the quality of the FRP−adhesive−concrete joint is to relate this quality to failure properties. Single [12] or double-lap joint [13] shear tests can provide pure mode II characterization of the bonded assembly, whilst adequately reproducing in-service loading conditions. These laboratory tests are widely used to investigate the influence of various parameters on the bond behavior, such as curing and ageing conditions of the polymer joint [14], substrate preparation [15], properties of the FRP material or bonded length [16]. Other test methods based on different loading modes are also used, but to a lesser extent [17,18]. 

**Figure 1:**
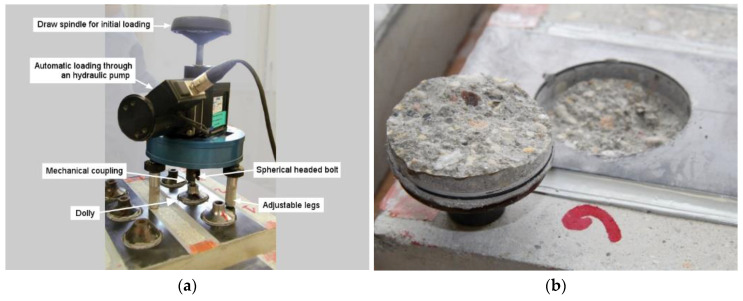
(**a**) Commercial pull-off tester; (**b**) typical cohesive failure in concrete substrate with pull-off strength of 5.9 MPa, demonstrating a sound bonded FRP system according to the standards.

Regarding field inspection, shear tests, like the shear tearing test [19,20] or the direct shear strip test [21], can be performed on site, but only when the FRP material can be pulled out along its plane, that is to say when it is located close to an edge of the strengthened structure and can be detached from the concrete surface. Le Roy et al. [22] have also developed a mobile device, which can conduct shear tests on FRP materials bonded to surfaces of various shapes (flat, concave or convex), but the machine remains quite heavy and its installation requires the following of a rigorous protocol. All the aforementioned tests need specific equipment and are thus not widely used in the field. Differently, the standardized direct tension pull-off test [23,24] is very popular for quantifying the adhesion level on control test panels. This test method, as shown on Figure 1a, consists of bonding a rigid metallic loading fixture called a dolly to the FRP surface and drilling a partial core around it through the bonded FRP and the adhesive layer into the concrete substrate with a specified range of depth (whose value depends on the recommendation in force). The dolly is then loaded perpendicularly to the FRP surface, until failure occurs at the so-called pull-off strength (ratio of the ultimate tensile load to the bonded surface of the dolly), revealing the weakest component of the system (see Figure 1b). A sound FRP-to-concrete bonded assembly shows a cohesive failure in the concrete substrate with a pull-off strength higher than a prescribed minimal value. Nevertheless, this partially destructive test, despite its popularity, has many drawbacks. First, test results show large scatter, which is inherent in the local singularities of concrete (presence of coarse aggregates underneath the test dolly), or in variations in the experimental conditions (influence of the depth of the partial core [25] or the effect of load eccentricity [26], for instance). Second, the measured strength may not be representative of the actual bond capacity, since the loading mode of the assembly (pure tension) is not representative of service conditions in the field. Third, the partial coring generates stress concentrations within the concrete material, and the results of the pull-off test are thus mostly governed by the mechanical properties of concrete, as this latter is often the weakest component of the bonded assembly. For all these reasons, inconsistencies may arise from test results, and their analysis may be difficult if no significant trend can be drawn [27].

In this context, there is a clear need to develop reliable NDT methods that are able to evaluate the level of adhesion (e.g., highly sensitive to parameters influencing the bond level) and suitable for use in the field. Such a method should satisfy the following requirements [25]: (a) simplicity of application, (b) ability to be rapidly deployed with minimal preparation, (c) providing easy to understand acceptance criteria with (d) a good level of repeatability and reproducibility.

The present study intends to develop an NDT method fulfilling all previous requirements. The proposed method is based on the standard pull-off test and consists of analyzing the load vs. displacement behavior of the composite−adhesive−concrete system in the linear elastic range [28,29]. The scope of this paper is to show the feasibility of this method for providing quantitative and discriminating data on the stiffness of the concrete/carbon FRP (CFRP) bonded assembly that can be further interpreted in terms of bond quality. The suggested NDT method is described in the next section.

## 2. Description of the NDT Method

First, it should be stressed that this feasibility study is restricted to the case of CFRP plate systems. Further research should be conducted in order to extend the results to other systems like fiber sheets bonded to the structure by wet lay-up process.

**Figure 2:**
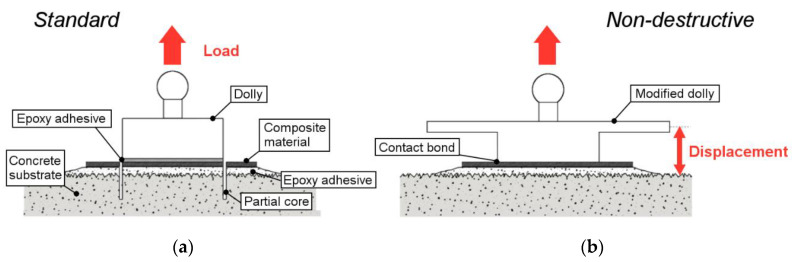
(**a**) Operating principle of the standard pull-off test and (**b**) operating principle of the modified ND pull-off test.

The principle of the NDT method is depicted on Figure 2b. As mentioned before, it is based on the standard pull-off test, but with three major differences: (1) the test zone is not isolated (i.e., there is no partial coring around the dolly in this case), (2) a displacement measurement is performed in addition to the load measurement (i.e., relative displacement between the upper plate of the dolly and the surface of the concrete block), and (3) the test is carried out in the linear elastic domain. From both measurements, a load vs. displacement curve can be plotted, whose slope will be called the bond stiffness or assembly stiffness.

The proposed NDT method relies on the following assumptions: the load vs. displacement behavior and the assembly stiffness are expected to be dependent on the Young’s modulus (E-modulus) of the polymer adhesive material, which is a key parameter regarding the performance of the strengthening FRP system. This E-modulus can be affected by the environmental conditions at the time of repair and may not reach its design value [30,31]. It is affected by curing conditions and ageing [14,32], and it also shows variations when the system is submitted to seasonal temperature variations (heatwaves or cold spells) since the glass transition temperature of cold-curing epoxies used in construction is rather low (generally around 50–60 °C), in which case early failures of the bonded assembly may occur [33]. For all these reasons, it is relevant to closely control the actual E-modulus of the adhesive layer in the bonded assembly, in order to assess the bond quality. From this perspective, the proposed nondestructive (ND) pull-off test appears as a very interesting tool.

The displacement should be measured at a suitable location on the concrete/FRP bonded assembly, so that it provides relevant information on the bond behavior. A differential displacement is also an interesting way to focus on the most interesting contributions. In the present study, a differential displacement between the dolly and the concrete surface was considered (Figure 2b). In addition to the E-modulus of the adhesive, several parameters influencing this displacement and the bond stiffness have been identified, namely the thickness of the adhesive, the elastic properties of the concrete substrate, the thickness of the concrete structure, the E-modulus and the thickness of the bonding agent between the dolly and composite plate. This issue is addressed in the developments. Otherwise, the deformation of the steel dolly is considered negligible and the contribution of the composite plate is supposedly well-known since it is a factory-pultruded material, hence no attention will be given to it. It should be pointed out that micron-level displacements are expected, and the overall instrumentation must be selected in accordance with this level of precision. 

In order to investigate the feasibility of the ND pull-off test, an experimental campaign was conducted, which is described in the following sections.

## 3. Experimental Program

The purpose of this experimental campaign is to check the capacity of the ND pull-off test to distinguish between different adhesive materials exhibiting various Young’s moduli. The variation range of the E-modulus is intended to simulate different levels of adhesion of the system, which could represent for instance different stages of degradation of the CFRP/concrete interface resulting from environmental exposure.

As an alternative approach, and to provide further information about the behavior of the system under ND pull-off loading, additional strain measurements were performed with distributed optical fiber sensors (DOFS) that were embedded in the adhesive layer.

### 3.1. Experimental Setup

#### 3.1.1. ND Pull-Off Test Setup

In order ensure a precise control of the experimental setup at the metrological level, to fully understand the mechanical phenomena at stake, and to identify the sensitive parameters, it was decided to carry out the tests with laboratory equipment instead of using a commercial pull-off tester. This implied various developments and protocols specific to the laboratory testing machine, which will be detailed in the following. However, it is important to keep in mind that a commercial device, like those manufactured by Ginger CEBTP (Elancourt, France) or Controls (Milan, Italy) among others, and as shown in Figure 1a, would be suitable for the test, provided this tester is equipped with a high precision load cell and a displacement measurement system. The field implementation of the test method should therefore be rather straightforward.

**Figure 3:**
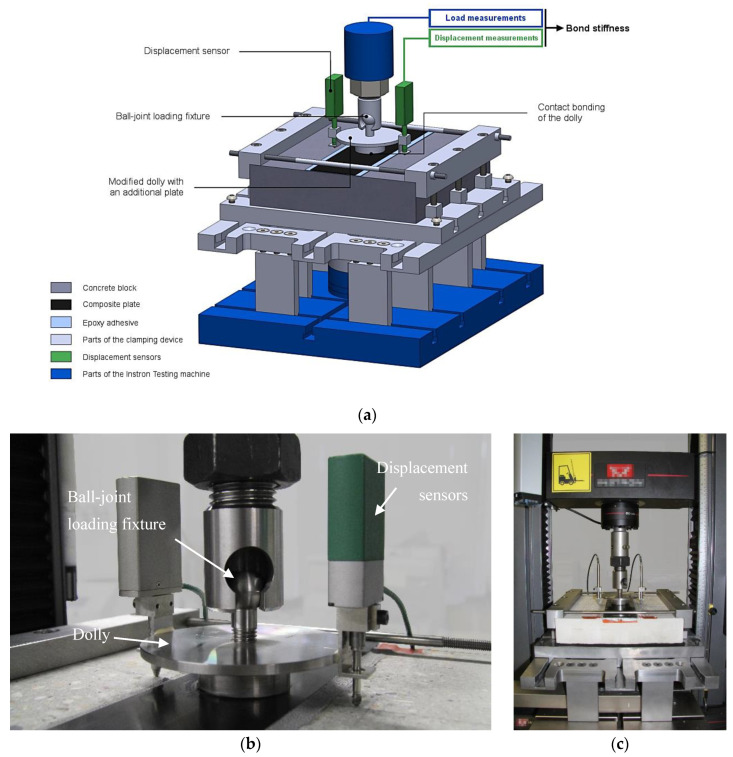
Modified ND pull-off test: (**a**) schematic view of a CFRP reinforced concrete specimen installed in the clamping device; (**b**) ball-joint loading fixture and displacement sensors fixed to the dolly; (**c**) clamping device installed on the testing machine.

ND pull-off tests were conducted on the 5969 universal testing machine (UTM) by Instron (Norwood, MA, USA) equipped with a load cell of capacity 50 kN. In order to firmly hold the concrete specimens, a clamping device, presented in Figure 3a,c, was designed using the commercial computed aided design software Solidworks by Dassault Systèmes (Vélizy-Villacoublay, France), and then machined by a precision manufacturer. This device mainly consists of two overlapping steel plates and allows two translational degrees of freedom for more flexibility in the specimens’ geometry. A finite element model (FEM) of the apparatus was computed to understand its behavior during the loading phase and estimate, for all bolts, the torques needed to ensure that the different parts were going to stay in contact, so that undesirable motion was minimized. The whole device was consequently assembled and installed on the UTM using a torque wrench and following a specific procedure aiming to ensure horizontality as much as possible.

As for the standard pull-off test, the load was applied to the dolly through a ball-joint fixture. For the present experimental campaign, the ball-joint system (a spherical headed bolt and a mechanical coupling) was supplied as the spare parts of a commercial pull-off tester. Special care was given to the centering of the spherical headed bolt into the coupling, in order to minimize undesirable flexion loading.

Special steel loading fixtures were produced, whose geometry was based on the shape of standard dollies. On top of a 50 mm diameter cylinder was added a 110 mm diameter and 5 mm thick plate, for a total height of 25 mm (see Figure 2b and Figure 3b). This plate made it possible to perform displacement measurements, which would not have been possible otherwise because of the bulk of the ball-joint coupling. Dollies were glued to the specimens using a contact cyanoacrylate adhesive. This latter contains a rubber component which improves the overall toughness, and exhibits increased flexibility and peel strength. Its low viscosity ensures a contact bond, which minimizes the tensile deformation of the bond line and its contribution to the measured displacement (this was previously identified as a parameter influencing the bond stiffness). The fixture time for a steel−CFRP interface (i.e., hardening time of the glue) was about 2–3 min, which gave enough time for positioning the dolly properly.

The displacement measurement between the dolly and the concrete surface was performed using the LE12/S high accuracy optical digital sensors developed by Solartron Metrology/Amatek (Elancourt, France), which provided consistent submicron measurement over a 12 mm displacement range. Two sensors were installed on each side of the system’s plane of symmetry, in order to get average values based on two displacement measurements. The two sensors were calibrated prior to the tests and presented expanded uncertainties of 0.9 µm and 0.6 µm (coverage factor of 2). They were attached to the sides of the dolly’s plate by means of steel rings glued with a 2-component fast curing methacrylate adhesive.

Load measurement was provided by the load cell of the universal testing machine. However, it was necessary to retrieve the load data with the acquisition system of the displacement sensors in order to synchronize both measurements. This was carried out by means of a specific input/output board and an analog-to-digital converter, both from the Orbit 3 system developed by Solartron Metrology/Amatek (Elancourt, France). This load acquisition system was also calibrated prior to the tests and presented an expanded uncertainty of 120 N.

All experiments were performed in a laboratory at a controlled temperature of 21 ± 2 °C. However, considering that the sensors were very sensitive to environmental parameters, a temperature and hygrometry probe was installed in the testing area.

#### 3.1.2. Optical Fiber Sensing Setup

As mentioned earlier, additional investigations were performed using DOFS instrumentation (Luna Technologies, Chicago, IL, USA), in order to collect complementary strain data and facilitate the analysis of the mechanical response of the system during the ND pull-off test.

DOFS is an innovative technology that allows long-range and continuous strain and temperature monitoring. A truly distributed optical fiber sensor (DOFS) consists of an interrogation unit paired with a standard optical fiber (OF) inserted into a protective coating, and embedded in or bonded to the monitored structure [34]. For the present study, optical frequency domain reflectometry (OFDR) was performed, based on the analysis of the Rayleigh backscattered light. According to the operating principle of the OFDR interrogation, a change in the longitudinal deformation state of the fiber, resulting from mechanical and/or thermal stresses, induces a change in the amplitude of the Rayleigh scattering, which is recorded. Hence, OFDR measurements are usually calibrated with respect to an initial state, and must be carried out while the structure is stable.

The Optical Backscatter Reflectometer OBR-4600 commercialized by Luna Technologies (Chicago, IL, USA) was used as the Rayleigh OFDR interrogator, and was connected to a single-mode polyimide-coated OF. It should be noted that this intermediate layer (primary polyimide coating) between the monitored structure and the OF sensor was so thin that the measured strain distribution could be directly considered as that of the structure [35].

Strain data were collected every 1 mm along the fiber, and resulted from an intercorrelation over a 10 mm gauge length, which helped to smooth the signal. 

### 3.2. Specimen

Different levels of adhesion were simulated using three commercially available epoxy resin adhesives (named Adhesives 1, 2 and 3), with Young’s moduli of 11,200, 4950 and 965 MPa, respectively, according to the manufacturer’s technical data sheets. It is worth noting that Adhesive 1 and Adhesive 2 are commercial systems intended for bonding CFRP plates onto concrete structures, while Adhesive 3 is a low modulus system that simulates an environmentally aged epoxy adhesive. The commercial brands of these adhesives are not specified here, due to confidentiality reasons. Unidirectional Sika Carbodur S CFRP laminates (Baar, Switzeland) were used. Their properties are listed in Table 1.

The experimental program consisted initially of testing eighteen concrete slabs strengthened with CFRP-laminates bonded using the three different adhesives (six specimens for each adhesive system). But some specimens (three for Adhesive 1 and one for Adhesive 3) were unfortunately damaged before being tested. One specimen for each adhesive system was picked out to perform DOFS strain measurements. The overall experimental program is summarized in Table 2.

**Figure 4:**
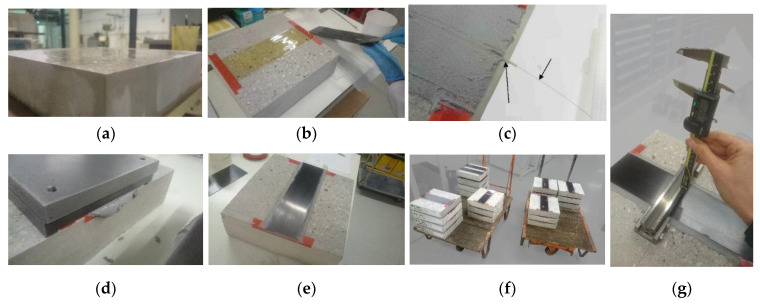
Specimen preparation: (**a**) ground concrete surface; (**b**) application of the adhesive by double-bonding; (**c**) insertion of the OF; (**d**) steel mass for pressing the bond line; (**e**) final aspect of a specimen; (**f**) set of specimens; (**g**) measurement of the thickness of the adhesive layer.

The 300 mm × 300 mm × 70 mm concrete slabs of type MC 0.45 were cast by the accredited manufacture company Rocholl GmbH, according to [36]. The maximum aggregate size was 10 mm. Three concrete batches were mixed for a total of thirty-six blocks (the rest of the blocks to be used in a durability study [28]). Due to the poor condition of the concrete surface, an extensive surface grinding process was achieved using a diamond wheel in a lapping machine, removing not only the laitance but also a small layer of the bulk concrete until the surface was planar at the scale of visual inspection. The aspect of the surface of a concrete slab after treatment is shown in Figure 4a.

The different steps of the specimen preparation are presented in Figure 4. For each specimen, a 300 mm long strip of composite plate was bonded at the center of the concrete slab. However, the edges of the concrete blocks were covered with a rubber tape of width 20 mm, in order to prevent edge effects. The total bonded length for the CFRP plate was therefore 260 mm. OF cutouts were inserted before joining both surfaces in the double-bonding process (where both concrete and composite plate surfaces were covered up with the epoxy adhesive before being pressed together), so the sensor was approximately placed at half-thickness of the adhesive layer. Thanks to the very small external diameter (160 µm) of the OF, this technique was considered as nonintrusive. The OF was later thermally welded to connectors compatible with the Rayleigh interrogator (Luna Technologies).

As mentioned earlier, the thickness of the adhesive layer was expected to affect the overall bond stiffness. Therefore, special care was taken to control the thickness as much as possible during specimen preparation. A steel mass with legs of height 3 mm was used to press the composite plate and the adhesive layer in order to ensure a repeatable adhesive joint thickness: by pressing the joint with the mass, the excess of adhesive is extracted until the adhesive layer reaches about 1.8 mm (3–1.2 mm corresponding to the thickness of the composite plate), as seen in Figure 4d. The final thickness in the central area of the plate was also measured with a caliper prior to the bonding of the dolly, as shown in Figure 4g. An average value of 1.7 ± 0.2 mm was obtained. These thickness values are used for the uncertainty quantification presented in Section 5.2.

### 3.3. Loading Procedure

**Figure 5:**
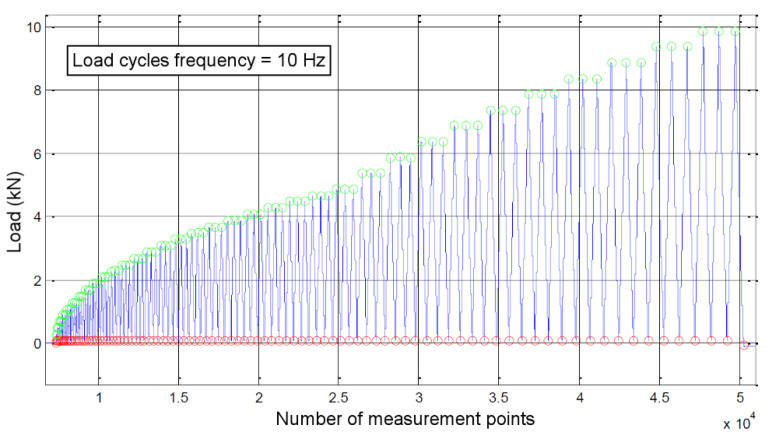
Evolution of the applied load (loading protocol).

A specific loading procedure was followed for all tests. Loading was displacement-controlled, the crosshead of the testing machine moving at an arbitrary speed of 0.3 mm/min. A cyclic protocol with a frequency of 10 Hz was applied, with a baseline at 200 N and the maximal load increasing every three cycles. An example of the loading protocol (up to 10 kN) can be visualized in Figure 5.

The three cycles per maximal load level helped in the recovery of redundant data for repeatability error estimation. The baseline of 200 N maintained the system under tension in case of mechanical backlash in the overall clamping device. The loading protocol was paused once per maximal load level to perform the DOFS strain measurements under stable conditions. Hence, a strain profile along the OF was obtained for each load level. 

## 4. Finite Element Modelling of the Test

A finite element analysis of the reinforced concrete block submitted to an ND pull-off test was carried out under the COMSOL Multiplysics software (version 5.2) in order to help with the analysis of the experimental results.

Both adhesive layers (between the dolly and the composite plate, and between the composite plate and the concrete surface) were modelled as linear springs, whose normal and tangent stiffnesses are derived from Hooke’s law:

$$k_{N}=\frac{1}{h}\cdot \frac{E}{1+\nu }\cdot \frac{1-\nu }{1-2\nu }\;\mathrm{and}\;k_{T}=\frac{1}{h}\cdot \frac{E}{2\left(1+\nu \right)}=\frac{G}{h}$$

E, ν and G being the elastic parameters (Young’s modulus, Poisson’s ratio, and shear modulus) of the adhesive layer and h its thickness.

A linear elastic behavior was adopted for each material. The material parameters were set to their experimentally measured values (see Section 5.2.1), or, in a first approach, to the values provided by the manufacturer in the case of CFRP plates (see Table 1). As for geometrical parameters, they were chosen in accordance with the test specimens’ dimensions.

**Figure 6:**
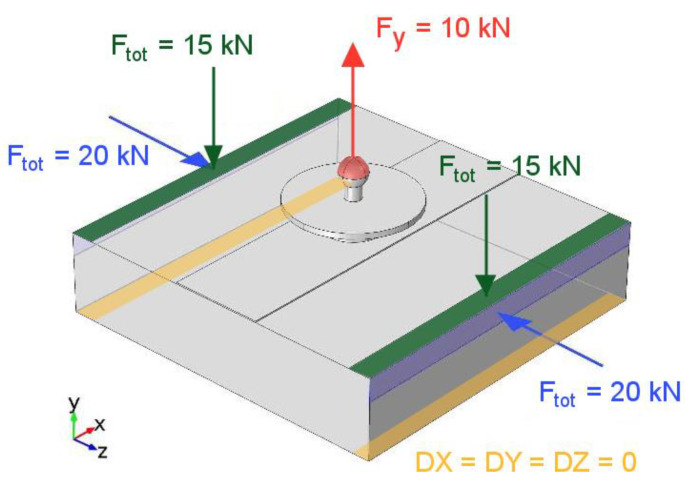
Applied boundary conditions for the simulated ND pull-off test.

The boundary conditions are summarized in Figure 6. The loads associated with clamping (depicted in blue and green) were calculated from the torques applied on bolts, which were experimentally measured with a torque wrench. All loads were uniformly applied as a pressure on the corresponding affected surfaces.

**Figure 7:**
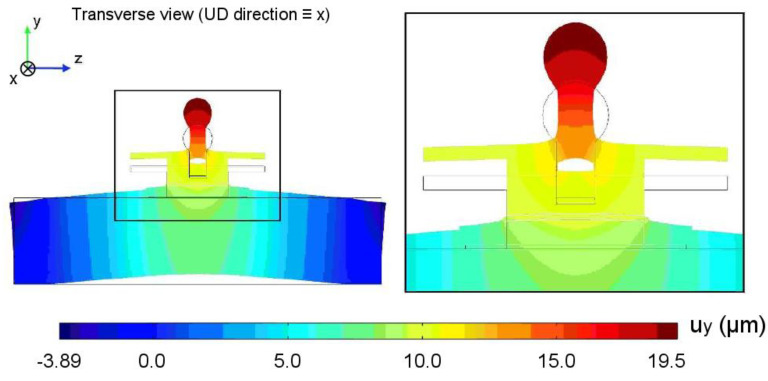
Normal displacement for a standard simulation with an applied pull-off load of 10 kN.

The typical response of the numerical system under pull-off loading in terms of normal displacement is displayed on the deformed geometry in Figure 7. A 10 kN load was applied to the spherical head. The concrete block exhibits a global flexural deformation. However, the response in the dolly area appears to be mainly governed by tension, which makes the test similar to its destructive standardized version regarding the mechanical behavior.

These modelling hypotheses are rather simple but were found effective to capture the experimental behavior, as the simulated ND pull-off load vs. displacement curves were found to match closely the experimental data. In order to get the best fit, the simulation was calibrated by tuning the E-modulus of the CFRP plate, as will be exposed in Section 5.2.2.

## 5. Results and Discussions

### 5.1. Load vs. Displacement Curve and Bond Stiffness

**Figure 8:**
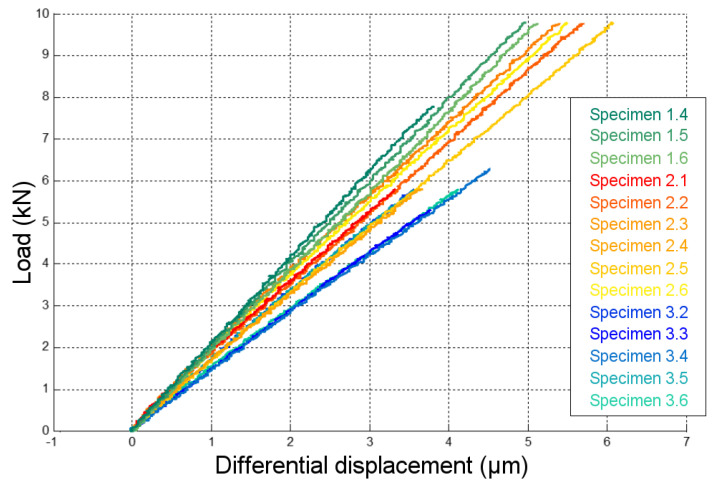
Load vs. displacement behavior acquired during the ND pull-off tests.

As a reminder, the displacement under consideration was the differential displacement between the test dolly and the concrete surface, as shown in Figure 3. The load vs. displacement curves of all fourteen exploitable specimens (see Table 2) are presented in Figure 8. Only the last load cycle of the elastic domain is shown. In these curves, the displacement is the mean of both sensor signals.

Values of the bond stiffness K, corresponding to the slope of the load vs. displacement curves, were also calculated through linear regression. Only the data until the limit load of each adhesive were considered (limit of the elasticity domain—see Section 5.3). Calculated values of the bond stiffness are reported in Table 3.

### 5.2. Uncertainty Analysis and Performance of the Test Method

To discuss the performance of the test method, both scatterings coming from the uncertain values of the sensitive parameters and experimental uncertainties have been taken into account. Sensitive parameters are defined as parameters for which a small variation in the statistic distribution has a large influence on the resulting bond stiffness.

#### 5.2.1. Estimation of Sensitive Parameters

All identified sensitive parameters were measured by means of different experimental methods. These methods are exposed in Table 4, together with the calculated means and standard deviations, where: E_concrete_ is the E-modulus of the concrete, h_concrete_ is the thickness of the concrete blocks, k_N,dolly-comp_ is the interface stiffness of the contact bond between the dolly and the composite plate, k_N,adh_ = E_adh_/h_adh_ is the interface stiffness of the adhesive layer between the composite plate and the concrete surface, h_adh_ being the thickness of this layer and E_adh_ its E-modulus. Depending on the situation, the standard deviation is either the uncertainty due to the measurement method, the repeatability error when the measurement is repeated over the same specimen, the scattering over all 18 specimens, or a quadratic sum of all errors. 

The E-modulus of concrete E_concrete_ was measured by transmission ultrasonic measurements and was then “converted” into a static E-modulus, following the method exposed in [37]. For the present study, two Acsys S1803 dry point contact transducers with a central frequency of 100 kHz were used, together with a Sofranel signal generator. The value of 62 GPa, derived from this method, appears to be very high and is most likely inconsistent, as the followed protocol turned out to be inappropriate. Considering that no specific concrete specimens were available to perform quasi-static tensile tests, this value was used in the present study.

The thickness of the concrete blocks h_concrete_ is identified as a sensitive parameter for the strengthened system under consideration, since the concrete blocks are rather thin and exhibit flexural deformation during the pull-off loading. Most likely this parameter would not influence the overall bond stiffness K during field inspections, as the real structures are expected to be much thicker. Further investigations could be performed in order to evaluate the lower limit of h_concrete_, for which this hypothesis still holds.

The interface stiffness k_N,dolly-comp_ was arbitrary estimated. As a contact bond, this interface stiffness is indeed expected to be very high compared to that of other interfaces in the system.

The transverse E-modulus of the composite plate, E_t,c_ is also a sensitive parameter. However, as composite plates are factory-pultruded materials, this parameter should not exhibit large scatter. A value of 15.4 GPa was found in [38].

#### 5.2.2. Reference Bond Stiffness

Although all sensitive parameters were discussed and experimentally measured, the FEM failed to reproduce the experimental bond stiffness K, when introducing mean values listed in Table 4: thus, either the numerical model is not representative, or one at least of the sensitive parameters was not properly estimated. The latter hypothesis was assumed true. This means that the numerical model needs further calibration, as it cannot be used to trace back the E-modulus of the adhesive. This was done by tuning the value of the E-modulus of the composite plate E_t,c._, which seemed a sound choice as no scattering of this parameter was expected from one specimen to the other. Calibration was achieved for Adhesive 1, when the associated mean experimental bond stiffness and the numerically predicted bond stiffness were equalized. In other words, Adhesive 1 provided a reference bond stiffness K_0_, and only differences with respect to this reference bond stiffness can be analyzed. The calibrated E-modulus of the composite plate was E_t,c_ = 4.5 GPa. 

As perfect measurements of all sensitive parameters can never be achieved, it is believed that the test results should always be interpreted in relation to a reference bond stiffness. 

#### 5.2.3. Experimental Uncertainties

An extended analysis was carried out to determine experimental uncertainties. All the following sources of errors were taken into account:

(1) Measurement uncertainties due to the load and displacement sensors,
(2) Errors arising from the postprocessing of the load vs. displacement data (square fit of uncertain data),
(3) Repeatability and reproducibility errors, which were evaluated by performing additional tests.

A final relative uncertainty of 2.7% on the measured bond stiffness K was computed.

#### 5.2.4. Performance of the Test Method: Minimal Detectable Value of the Adhesive E-Modulus

Once the numerical model was calibrated, the probability distributions of the irrelevant sensitive parameters (all except E_adh_) could be converted into a distribution of the reference bond stiffness K_0_. Normal distributions were arbitrarily assumed for all sensitive parameters. By adding the effects of the sensitive parameters in the most conservative way, it was found that the reference stiffness K_0_ is normal distributed with a standard deviation of 0.22 × 10^9^ N/m.

As experimental uncertainties affect both the reference and the investigated system’s bond stiffness measurements, it should be taken into account twice. By quadratic summing errors coming from uncertain sensitive parameters and from experimental uncertainties, it can be claimed that a variation (decrease) in K with respect to K_0_ can be inferred to an actual decrease in the adhesive E-modulus:

(1) With a 67% confidence level for a variation of 0.23 × 10^9^ N/m
(2) With a 99% confidence level for a variation of 0.46 × 10^9^ N/m

**Figure 9:**
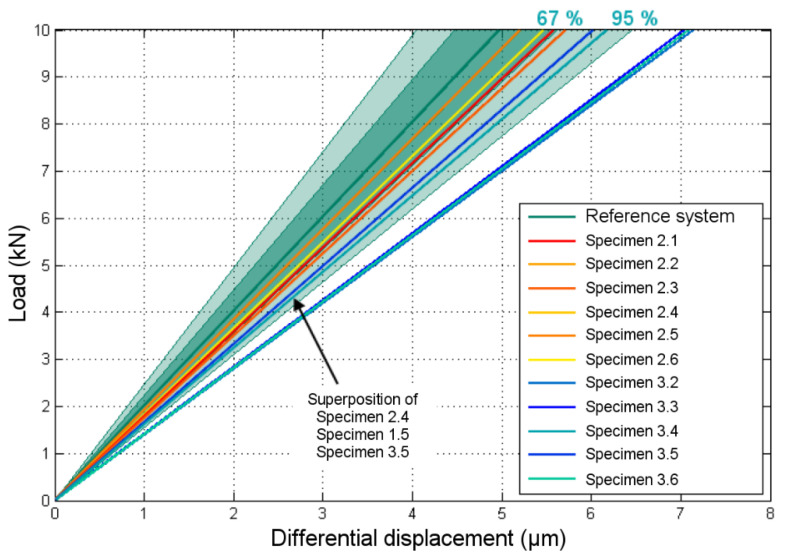
Load vs. displacement curves for specimens with Adhesive 2 and Adhesive 3, and probability distributions for the reference system.

These results are schematically represented in Figure 9. Thanks to the FEM, they can be translated into a minimal detectable E-modulus of the adhesive: providing that the reference system is Adhesive 1 with E-modulus of 11.2 GPa, a loss of bond stiffness associated to an adhesive with E-modulus of 4.0 GPa can be detected with a 67% confidence level, and of 2.2 GPa with a 95% confidence level.

A quick estimation of the minimal detectable E-modulus of the adhesive for real field testing conditions was performed by reconsidering the probability distributions of the sensitive parameters. A 400 mm-thick concrete structure was considered. The most critical change concerned the concrete deformation mode that passed from a flexure dominant mode to a tension dominant mode. Together with a decrease in the concrete E-modulus (whose value was considered too high, as previously underlined in Section 5.2.1), the weight of the concrete contribution to the overall bond-stiffness became more important, which degraded the performance of the test method. A minimal detectable adhesive E-modulus of 1.1 GPa was found for a 95% confidence level.

### 5.3. Determination of the Limit Load for the ND Test

**Figure 10:**
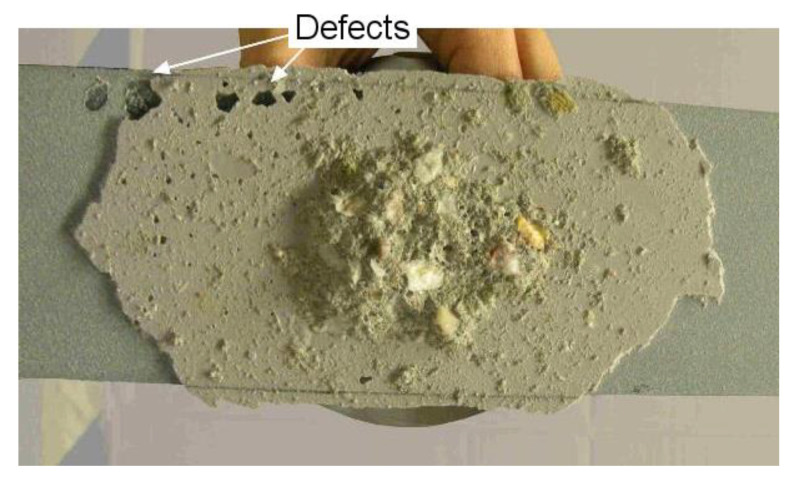
Example of a fracture aspect after complete debonding of the composite plate.

A precise analysis of the failure behavior was carried out, in order to determine the limit load that specimens should not exceed to ensure the ND nature of the test method. All specimens were brought to failure so the limit of the elasticity domain and the failure modes can be investigated. All these specimens but one (with Adhesive 3) exhibited a brittle cohesive fracture in the concrete material underneath the dolly, accompanied by a loud, dull sound. This confirmed that the loading conditions were very similar to those encountered with the standard destructive pull-off test. If the specimen was further loaded, the crack propagated following first the concrete/adhesive interface, and then the adhesive/composite interface. A fracture aspect after complete debonding of the composite plate can be observed on Figure 10.

For some specimens, few bond voids were exposed after complete debonding, but they did not seem to influence the overall fracture behavior nor the bond stiffness, most probably because they were small and located by the edges of the composite plate. Whether a large void located underneath the dolly impacts the results or not could be further investigated. However, it did not appear to be of paramount importance, as voids could be detected by other ND techniques such as infrared thermography in order to prevent gluing the dolly onto unsound areas. 

It should be mentioned that a few specimens failed first by debonding at the composite−dolly interface, but could be further tested after re-bonding of the dolly. However, the cyanoacrylate adhesive used to achieve a contact bond between the dolly and the composite plate provided satisfying results, as this “first” failure mode remained scarce.

Determined values of elasticity limits and failure loads are both reported in Table 5. It should be pointed out that the elastic limit was lower than the failure load, but it is believed that nonlinearities arose due to misalignments or horizontality defects in the mechanical device and/or in the joint “layers” and not to the constitutive behavior of the system. This hypothesis is supported by the results of the DOFS measurements, which exhibited a linear behavior beyond the elasticity limit discussed here, as will be exposed in Section 5.5. Nonetheless, the nonlinearities were partially or totally balanced when the signal of both displacement sensors was averaged. This stresses the relevance of multiple displacement measurements.

For all these reasons, the values of limit load were chosen in relation to the failure loads. The recommended limit loads for the ND test are reported in Table 5. 

### 5.4. Recommended Testing Procedure for on-Site Inspection

Although the validation of the ND pull-off test was performed in laboratory conditions in the present study, the test method was intended to be deployed in field conditions. From previous observations and discussions, an on-site test procedure for the monitoring of the ageing of the bond quality of an FRP strengthening system can be outlined:

(1) Small, strengthened test panels (about 200 mm × 200 mm) should be bonded next to the strengthening system to be monitored at the time of installation T_0_. The total amount of test panels should be planned in relation to the target number of monitoring terms;
(2) Multiple displacement sensors should be used and disposed (i.e., attached to the dolly) consistently with the symmetry of the system, so the displacement data can be averaged;
(3) At each term, a series of tests should be performed until failure on the dedicated test panels in order to assess the limit load for the ND pull-off test. An 80% rate of the minimal failure load is recommended;
(4) At each term, additional small test panels should be installed using the reference adhesive system. Special care should be given to find a location of similar concrete conditions. The reference panels should be cured under optimal conditions. The ND pull-off test on these panels provides the reference bond stiffness K_0_;
(5) The ND pull-off test should be performed on the monitored strengthening system to assess its bond stiffness for the current term;
(6) Using the methodology based on the calibrated FEM followed in paragraph 5.2, the change in bond stiffness which can be inferred with a high confidence to a degradation of the E-modulus can be announced. If the bond stiffness K of the monitored system appears to be lower than this critical change, the associated E-modulus of the adhesive should be traced back with the numerical model.

If the estimated E-modulus of the adhesive system is considered too low according to the available knowledge on the adhesive material’s behavior and performance, then the strengthened system should undergo maintenance.

### 5.5. Results of the Optical Fiber Strain Measurements

**Figure 11:**
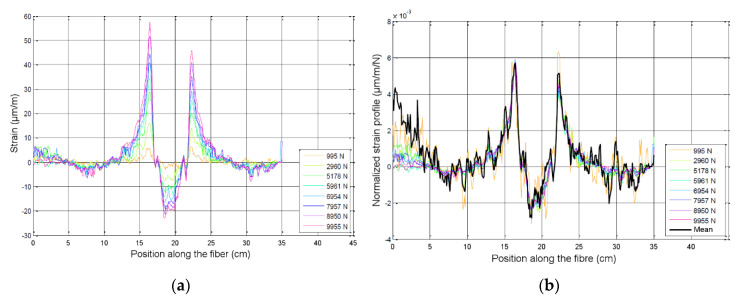
(**a**) DOFS strain profiles at increasing load levels for the joint of Adhesive 1; (**b**) normalized strain profiles.

As explained in Section 3.1.2, DOFS measurements were performed in the epoxy adhesive layer at different pull-off loads, so that the evolution of the strain profile along the fiber with increasing load levels could be observed. As can be seen in Figure 11a, the profiles exhibited progressive strain concentrations at locations of the joint corresponding to the dolly’s edge. Besides, compressive strain was recorded under the area where the dolly was bonded, which was consistent with the loading conditions.

**Figure 12:**
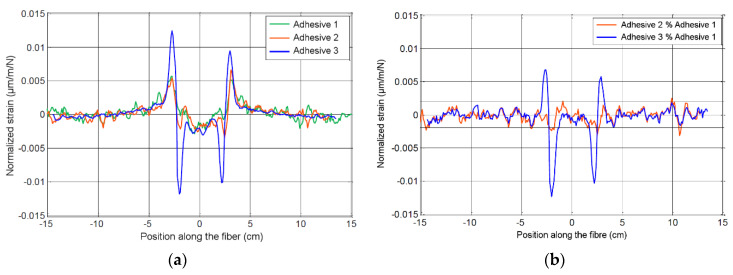
(**a**) Normalized strain profiles (mean signal averaged on all profiles obtained at the various load levels) for joints of all adhesive systems; (**b**) comparison of the normalized strain profiles for joints of Adhesives 2 and 3 with respect to the profile of Adhesive 1.

The strain profiles collected for the joints of Adhesive 1 and Adhesive 2 exhibited a higher noise level than that of Adhesive 3 (see Figure 12a), which could be explained by the larger shrinkage of these adhesives (due to their specific admixtures formulation) during the curing process, which pinched the optical fiber. 

It should be first pointed out that the DOFS profiles exhibited nonzero strain along a length much shorter than the total bonded length (in the longitudinal direction of the plate), so it can be affirmed that the composite plate was glued on a sufficiently long length. The size of the area mechanically affected by the ND pull-off loading was rather small (about 200 mm for all adhesive systems), which confirmed that the ND pull-off test could be performed on small test panels.

As illustrated on Figure 11b for the joint of Adhesive 1, the strain profiles matched very well when normalized by the load level, which tended to confirm that the behavior remained linear until the brittle failure of the specimen occurred. The same observations were made for joints of Adhesives 2 and 3.

A comparison between normalized strain profiles of specimens with the three adhesives is shown in Figure 12a. The profile of the joint of Adhesive 3 can be clearly distinguished from those of the two other systems. This was even more obvious when the normalized strain profile of Adhesive 1 was subtracted from the profiles of Adhesive 2 and Adhesive 3, as depicted in Figure 12b. The response of Adhesive 3 clearly emerged out of the noise, which was consistent as the deformation within the adhesive layer was much higher for Adhesive 3 (of lowest E-modulus) than for Adhesive 1 (of highest E-modulus). 

From these results, it appears that the strain profiles measurement under a ND pull-off load by means of a DOFS embedded in the adhesive layer is an interesting alternative and complementary approach to quantify the bond quality of an FRP strengthening system, as a decrease in the E-modulus of the adhesive induces a detectable change in the strain profile, and should be investigated further. In the present study, a decrease of 10 GPa with respect to Adhesive 1, considered as a reference system, could be detected, which is similar to the performance of the ND pull-off test itself. A precise quantification of uncertainties coming from sensitive parameter distributions and from repeatability and reproducibility analysis could facilitate the assessment of the detection performance, in a similar fashion to that in Section 5.2. 

## 6. Conclusions and Outlooks

The proposed ND pull-off test method is based on the well-known and standardized destructive pull-off test, but without any drilling operation and considering a displacement measurement. Together with the load data, it makes it possible to collect the load vs. displacement behavior of the CFRP−concrete strengthening system, whose slope is called the bond stiffness of the system.

In the present study, the features of the test method were examined on laboratory equipment thanks to a specific mechanical clamping device, and the performance of the test method was estimated by performing a precise analysis of all uncertainties arising from sensitive parameters and from experimental measurements. The following results have been obtained:

(1) The load vs. displacement response of the joint appears to hold information about the E-modulus of the adhesive layer between the composite plate and the concrete surface, which in turn is related to the bond quality of the system;
(2) The results of the test method appear to be most relevant when compared to a reference system tested under the same conditions;
(3) The performance of the test method is expressed in terms of a minimal detectable E-modulus of the adhesive layer, given the E-modulus of the reference system (in other words, in terms of a minimal detectable change in E-modulus). For a reference E-modulus of 11.2 GPa, a degradation down to 4.0 GPa will be detected with a 67% confidence level, and down to 2.2 GPa with a 95% confidence level;
(4) The loading conditions are very similar to those of the standard destructive pull-off test, and the specimens exhibit a brittle behavior when tested until failure. Hence, it can be considered performing the ND test up to 80% of the failure load;
(5) The test method could be easily adapted for field inspection as it is designed with the same loading ball-joint fixture as a commercial pull-off tester;
(6) The suggested measurement chain consists of load measurements synchronized with two high accuracy and long range optical digital sensors collecting the differential displacements between the test dolly bonded to the composite surface and the concrete surface. It is perfectly suitable for the purpose of the test and could be easily transferred in the field, provided that the load cell of the commercial pull-off tester is accurate enough;
(7) Averaging multiple displacement measurements appears to be relevant to offset alignment or horizontality defects;
(8) Additional distributed strain measurements by means of DOFS embedded in the adhesive layer provide useful information to understand the behavior of the system under pull-off loading. It also happens to be a promising alternative approach to quantify the bond quality as the strain profiles depend on the E-modulus of the adhesive.

Future work should be dedicated to investigation of the following key aspects:

1. The E-modulus of the concrete material highly impacts the bond stiffness of the joint and its influence should be carefully assessed and quantified. It could be interesting to address whether an ND pull-off test on the bare concrete surface could provide a background value to normalize the test results;
2. The ageing behavior of the adhesive joint should be monitored with the proposed test method in the context of an experimental durability program in order to assess the performance of the test method with an environmentally degraded E-modulus. Note that the failure load of the system is expected to decrease during ageing, while the system response might exhibit a more ductile behavior. Hence, the limit load that should not be exceeded during the ND test is expected to decrease at each inspection term. This will reduce the set of data available for the determination of the bond stiffness;
3. The field application should be broader to encompass other usual practical cases, in particular systems strengthened by wet lay-up processes and/or with multiple composite layers.

## Figures and Tables

**Table 1:** Typical properties of the CFRP plates used in the present study.

Description	Thickness	Width	Fiber Content	Tensile Properties (EN 2561)		
Pultruded unidirectional (0°) CFRP plate	1.2 mm	80 mm	>68%	Modulus: 165,000 MPa	Tensile strength: 2900 MPa	Strain at failure: >1.80%

**Table 2:** Experimental program.

Specimen ID	Adhesive 1			Adhesive 2						Adhesive 3				
	1.4	1.5	1.6	2.1	2.2	2.3	2.4	2.5	2.6	3.2	3.3	3.4	3.5	3.6
Displacement Data	🗸	🗸	🗸	🗸	🗸	🗸	🗸	🗸	🗸	×	🗸	🗸	🗸	🗸
OF Strain Measurement	×	🗸	×	×	×	×	×	×	🗸	×	×	×	×	🗸

**Table 3:** Values of the bond stiffness K calculated by linear regression of the load vs. displacement curves.

Specimen ID	Adhesive 1			Adhesive 2						Adhesive 3				
	1.4	1.5	1.6	2.1	2.2	2.3	2.4	2.5	2.6	3.2	3.3	3.4	3.5	3.6
Bond stiffness (×10^9^ N/m)	2.04	1.99	1.94	1.79	1.62	1.75	1.62	1.92	1.83	1.40	1.42	1.62	1.66	1.41
Mean (×10^9^ N/m)	1.99			1.76						1.50				
Standard deviation (×10^9^ N/m)	0.03			0.12						0.13				

**Table 4:** Sensitive parameters: means, standard deviations and measurement methods.

Sensitive Parameter		Mean	Standard Deviation	Measurement Method
E_concrete_		62 GPa	7 GPa	Ultrasonic measurement of the dynamical E-modulus
h_concrete_		67.6 mm	1.7 mm	Ruler
k_N,dolly-comp_		2.0 × 10^13^ Pa/m	1.0 × 10^13^ Pa/m	None (arbitrary values)
k_N,adh_ = E_adh_/h_adh_	Adhesive 1	1.61 × 10^13^ Pa/m	0.7 × 10^13^ Pa/m	Measurement of h_adh_ with a caliper * (see Figure 4g) Measurement of E_adh_ by direct tensile tests on bulk adhesive coupons
	Adhesive 2	0.57 × 10^13^ Pa/m	0.11 × 10^13^ Pa/m	
	Adhesive 3	0.17 × 10^13^ Pa/m	0.07 × 10^13^ Pa/m	

* The maximum permissible error (MPE) was not taken into account in the obtained standard deviation.

**Table 5:** Experimental values of the elastic limits and failure loads of the various specimens, and recommended limit loads.

Specimen ID	Adhesive 1			Adhesive 2						Adhesive 3				
	1.4	1.5	1.6	2.1	2.2	2.3	2.4	2.5	2.6	3.2	3.3	3.4	3.5	3.6
F_elas_ (kN)	8	12	14	6	10	12	7	12	10	10	10	6	10	10
F_ult_ (kN)	13.4	13.8	16.1	10	12	14.2	10	16	13.3	12	14	10	14	14.2
Limit load (kN)	10 kN			8 kN						8 kN				

## References

[1] Cascardi A., Leone M., Aiello M.A. (2020). Transversal joining of multi-leaf masonry through different types of connector: Experimental and theoretical investigation. Constr. Build. Mater..

[2] Hai N.D., Mutsuyoshi H., Asamoto S., Matsui T. (2010). Structural behavior of hybrid FRP composite I-beam. Constr. Build. Mater..

[3] Drosopoulos G.A., Stavroulakis G.E., Massalas C.V. (2007). FRP reinforcement of stone arch bridges: Unilateral contact models and limit analysis. Compos. Part B.

[4] Sansalone M., Street W. (1998). The impact-echo method. NDT.net.

[5] Ribolla E.L.M., Hajidehi M.R., Scimemi G.F., Spada A., Giambanco G. (2016). Assessment of bonding defects in FRP reinforced structures via ultrasonic technique. Chall. J. Struct. Mech..

[6] Sun R., Sevillano E., Perera R. (2015). Debonding detection of FRP strengthened concrete beams by using impedance measurements and an ensemble PSO adaptive spectral model. Compos. Struct..

[7] Gholizadeh S. (2016). A review of non-destructive testing methods of composite materials. Procedia Struct. Integr..

[8] Taillade F., Quiertant M., Benzarti K., Aubagnac C. (2011). Shearography and pulsed stimulated infrared thermography applied to a ND evaluation of FRP strengthening systems bonded on concrete structures. Constr. Build. Mater..

[9] Qiu Q., Lau D. Use of laser reflection technique for defect detection in CFRP concrete systems. Proceedings of the Nondestructive Characterization and Monitoring of Advanced Materials, Aerospace, and Civil Infrastructure.

[10] Taillade F., Quiertant M., Benzarti K., Aubagnac C., Moser E. (2011). Shearography applied to the non destructive evaluation of bonded interfaces between concrete and CFRP overlays: From the laboratory to the field. Eur. J. Environ. Civ. Eng..

[11] Qiu Q. (2020). Imaging techniques for defect detection of fiber reinforced polymer-bonded civil infrastructures. Struct. Control. Health Monit..

[12] Chataigner S., Caron J.F., Benzarti K., Quiertant M., Aubagnac C. (2011). Use of a single lap shear test to characterize composite-to-concrete or composite-to-steel bonded interfaces. Constr. Build. Mater..

[13] Quiertant M., Benzarti K., Schneider J., Landrin F., Landrin M., Boinski F. (2017). Effects of Ageing on the Bond Properties of Carbon Fiber Reinforced Polymer/Concrete Adhesive Joints: Investigation Using a Modified Double Shear Test. J. Test. Eval..

[14] Benzarti K., Chataigner S., Quiertant M., Marty C., Aubagnac C. (2011). Accelerated ageing behaviour of the adhesive bond between concrete specimens and CFRP overlays. Constr. Build. Mater..

[15] Iovinella I., Prota A., Mazzotti C. (2013). Influence of surface roughness on the bond of FRP laminates to concrete. Constr. Build. Mater..

[16] Ferrier E., Quiertant M., Benzarti K., Hamelin P. (2010). Influence of the properties of externally bonded CFRP on the shear behavior of concrete/composite adhesive joints. Compos. Part. B..

[17] Gartner A., Douglas E.P., Dolan C.W., Hamilton H.R. (2011). Small Beam Bond Test Method for CFRP Composites Applied to Concrete. J. Compos. Constr..

[18] Karbhari V.M., Navada R. (2008). Investigation of durability and surface preparation associated defect criticality of composites bonded to concrete. Compos. Part. Appl. Sci. Manuf..

[19] CNR (2013). Guide for the Design and Construction of Externally Bonded FRP Systems for Strengthening Existing Structures—Report CNR-DT 200 R1/2013.

[20] Nuti C., Santini S., Sguerri L. In situ Shear-tearing test for the quality control on FRP-to Concrete bonded joints. Proceedings of the 7th International Conference on Fiber Reinforced Polymer (FRP) Composites in Civil Engineering (CICE 2014).

[21] Petersen C.G., Poulsen E. (2001). In-Situ Quality Control of Structural Strengthening by Epoxy-Bonded CFRP Strips. Personal Communication by Germann Instruments. http://www.germann.org/TestSystems/sMASH/smash_14.pdf.

[22] Le Roy C., Aubagnac C., Flety A., Chataigner S., Benzarti K., Quiertant M. Mobile device to perform on-site single lap shear test on FRP bonded on RC structures. Proceedings of the 9th International Conference on Fibre-Reinforced Polymer (FRP) Composites in Civil Engineering (CICE 2018).

[23] ASTM (2015). Standard Test Method for Pull-Off Strength for FRP Laminate Systems Bonded to Concrete Substrate.

[24] EN 1542 (1999). Products and Systems for The Repair and Protection of Concrete Structures.

[25] Eveslage T., Aidoo J., Harries K.A., Bro W. (2010). Effect of Variations in Practice of ASTM D7522 Standard Pull-Off Test for FRP-Concrete Interfaces. J. Test. Eval..

[26] Courard L., Garbacz A., Moczulski G. Evaluation of the effect of load eccentricity on pull-off strength. Proceedings of the 2nd International Conference on Concrete Repair, Rehabilitation and Retrofitting (ICCRRR).

[27] Mata O.R., Atadero R.A. (2014). Evaluation of Pull-Off Tests as a FRP–Concrete Bond Testing Method in the Laboratory and Field. Pract. Period. Struct. Des. Constr..

[28] Billon A. (2016). Méthode D’évaluation ND de la Qualité du Collage des Composites de Renforcement Pour le Génie Civil. Ph.D. Thesis.

[29] Billon A., Taillade F., Quiertant M., Henault J.M., Maurin R., Benzarti K. Assessment of the bond quality between concrete and FRP strengthening systems using a novel nondestructive test. Proceedings of the 8th International Conference on FRP Composites in Civil Engineering (CICE 2016).

[30] Benedetti A., Fernandes P., Granja J.L., Sena-Cruz J., Azenha M. (2016). Influence of temperature on the curing of an epoxy adhesive and its influence on bond behaviour of NSM-CFRP systems. Compos. Part. B.

[31] Moussa O., Vassilopoulos A.P., de Castro J., Keller T. (2012). Early-age tensile properties of structural epoxy adhesives subjected to low-temperature curing. Int. J. Adhes. Adhes..

[32] Karbhari V.M., Ghosh K. (2009). Comparative durability evaluation of ambient temperature cured externally bonded CFRP and GFRP composite systems for repair of bridges. Compos. Part. Appl. Sci. Manuf..

[33] Ferrier E., Rabinovitch O., Michel L. (2015). Mechanical behavior of concrete–resin/adhesive–FRP structural assemblies under low and high temperatures. Constr. Build. Mater..

[34] Billon A., Hénault J.M., Quiertant M., Taillade F., Khadour A., Martin R.P., Benzarti K. (2015). Qualification of a distributed optical fiber sensor bonded to the surface of a concrete structure: A methodology to obtain quantitative strain measurements. Smart Mater. Struc..

[35] Hénault J.M., Quiertant M., Delepine-Lesoille S., Salin J., Moreau G., Taillade F., Benzarti K. (2012). Quantitative strain measurement and crack detection in RC structures using truly distributed fiber optic sensing system. Constr. Build. Mater..

[36] EN1766 (2017). Products and Systems for the Protection and Repair of Concrete Structures.

[37] Haach V.G., Marrara Juliani L., Ravanini Da Roz M. (2015). Ultrasonic evaluation of mechanical properties of concretes produced with high early strength cement. Constr. Build. Mater..

[38] Castellano A., Foti P., Fraddosio A., Marzano S., Piccioni M.D. (2014). Mechanical characterization of CFRP composites by ultrasonic immersion tests: Experimental and numerical approaches. Compos. Part B.

